# Evaluation and commissioning of a surface based system for respiratory sensing in 4D CT

**DOI:** 10.1120/jacmp.v12i1.3288

**Published:** 2010-12-04

**Authors:** Maria Francesca Spadea, Guido Baroni, David P. Gierga, Julie C. Turcotte, George T.Y. Chen, Gregory C. Sharp

**Affiliations:** ^1^ Department of Experimental and Clinical Medicine Magna Graecia University viale Europa Catanzaro Italy; ^2^ Bioengineering Department Politecnico di Milano University Milano Italy; ^3^ Department of Radiation Oncology Harvard Medical School and Massachusetts General Hospital Boston 02114 MA USA

**Keywords:** 4D CT, respiratory motion, surface imaging, multi sensor analysis

## Abstract

The purpose of this study is to assess the temporal and reconstruction accuracy of a surface imaging system, the GateCT under ideal conditions, and compare the device with a commonly used respiratory surrogate: the Varian RPM. A clinical CT scanner, run in cine mode, was used with two optical devices, GateCT and RPM, to detect respiratory motion. A radiation detector, GM‐10, triggers the X‐ray on/off to GateCT system, while the RPM is directly synchronized with the CT scanner through an electronic connection. Two phantoms were imaged: the first phantom translated on a rigid plate along the anterior–posterior (AP) direction, and was used to assess the temporal synchronization of each optical system with the CT scanner. The second phantom, consisting of five spheres translating 3 cm peak‐to‐peak in the superior–inferior direction, was used to assess the quality of rebinned images created by GateCT and RPM. Calibration assessment showed a nearly perfect synchronization with the scanner for both the RPM and GateCT systems, thus demonstrating the good performance of the radiation detector. Results for the volume rebinning test showed discrepancies in volumes for the 3D reconstruction (compared to ground truth) of up to 36% for GateCT and up to 40% for RPM. No statistical difference was proven between the two systems in volume sorting. Errors are mainly due to phase detection inaccuracies and to the large motion of the phantom. This feasibility study assessed the consistency of two optical systems in synchronizing the respiratory signal with the image acquisition. A new patient protocol based on both RPM and GateCT will be soon started.

PACS number: 87

## I. INTRODUCTION

The importance of dealing with respiratory motion issues^(^ ^1^ ^,^ ^2^ ^,^ ^3^ ^)^ both during the planning and dose delivery, is driving the development of very sophisticated tools to account for breathing physiology. Different methods^(^ ^4^ ^,^ ^5^ ^,^ ^6^ ^,^ ^7^ ^,^ ^8^ ^)^ have been proposed to acquire the patient respiration movements during the simulation session for 4D CT reconstruction. One common method of quantifying 4D CT images, utilizes the Varian RPM system to monitor patient respiration and allow retrospective resorting of the 4D CT images. In general, while 4D CT is useful in visualizing respiratory tumor motion, there can be inaccuracies in phase detection that generate artifacts in reconstructed volumes. The GateCT system, commercialized by Vision RT (London, UK), has been recently installed in the simulation room of the Radiation Oncology Department at Massachusetts General Hospital (Boston). This system features 3D surface capture capabilities together with surface patch tracking to allow retrospective 4D CT reconstruction. A potential advantage of this device is that it captures surface images that can be used to verify patient setup, as well as to monitor intrafraction motion over a region of interest. Our current 4D CT protocol relies on RPM system^(^ ^8^ ^)^ where a single marker is tracked by means of an infrared (IR) camera.

In this work, we present a multisensory analysis aiming at evaluating the consistency of the GateCT and RPM systems in 4D volume resorting. Our goal is to test and commission the GateCT device for simultaneous use with RPM, so that we may perform direct comparisons between the two devices. We first evaluated the quality of the synchronization between image acquisition and respiratory waveforms. Secondly, we assessed the accuracy in reconstructing moving spheres with known 3D volumes over 10 respiratory phases by the two systems. Lastly, we evaluated the interference of the two devices in terms of light projection.

## II. MATERIALS AND METHODS

The GateCT (GCT) system is an extension of Align RT^(^ ^9^ ^,^ ^10^ ^)^ that was designed for patient setup assessment. A single camera is mounted on the ceiling of the CT room in line with the CT couch for markerless patient surface tracking. Sampling frequency depends on the size of the detected area used to generate respiratory signal. In this work, we set the default size (20×20mm) leading to a sampling rate of 16 Hz. The motion of the CT couch is taken into account by the system via software, on the basis of specific CT parameters. The RPM IR camera (Varian, Palo Alto, CA) is mounted on the CT couch and has a sampling frequency of 30 Hz. Both systems project light in the visible or near‐infrared spectrum to allow accurate optical tracking. The scanner used in this study is the GE LightSpeed RT16 scanner (GE Healthcare, Waukesha, WI).

Both devices are synchronized with the image acquisition, but through different mechanisms. The RPM system has an electrical connection with the GE gantry, while the GCT system uses an external radiation detector, the Blackcat GM‐10 (Blackcat, Westminster, MD). It is possible to synchronize the GE with GCT electronically, instead of using the Blackcat detector, but it is not possible to simultaneously connect both devices with the GE scanner. This configuration allows us to use both devices at the same time, for the purpose of performing a head‐to‐head comparison.

### A. Experiment 1: Synchronization

**Figure 1:**
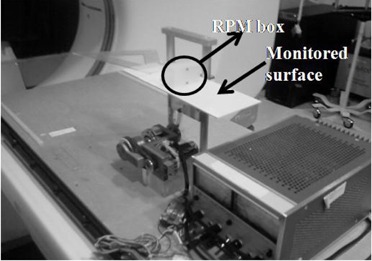
Experimental setup for the assessment of the calibration of CT scanner and optical systems for 4D image reconstruction.

The experimental setup is shown in Fig 1. A rigid object (a set square) was mounted on plate, a horizontal plate, which moved quasi‐sinusoidally in the anterior–posterior direction with a period of 3.5 seconds. The moving plate was imaged by the CT scanner using the GE cine‐mode 4D CT protocol, and simultaneously imaged by both the RPM system, and the GCT system. A region of interest on the plate surface was monitored by the GateCT system, and we will refer to this signal as *gct_amp*. The rpm box was placed on the same plate, and its signal will be identify as *rpm_amp*.

The parameters used during image acquisition were the following: image reconstruction on a full gantry rotation (0.5 sec), 0.3 sec as cine time interval and 2.5 mm as slice thickness, 8 slices per couch position (cp). A total of 1120 images were obtained on 10 cp, thus resulting in 14 eight‐slice chunks per cp.

**Figure 2:**
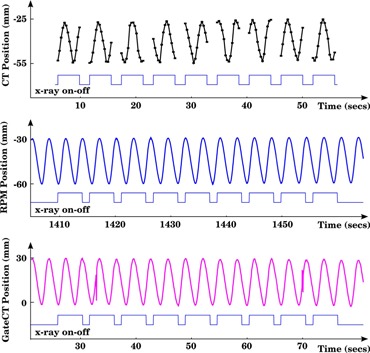
Motion amplitude of the square set measured on the images (ground truth) and detected by RPM and GCT. The object extended over nine couch positions, from the second to the tenth one, as can be noted from the ground truth pattern. Both rpm‐amp and gct‐amp present a time offset with respect to the scanner due to different clocks of the systems. The offset was computed by aligning the X‐ray on/off pulses.

Next, we measured the position of the square set on each slice of the unsorted image dataset, and related it to the midscan time (assumed as acquisition time) extracted from the DICOM header. The obtained signal is taken as the *ground truth* of the motion amplitude (see Fig. 2, upper panel). To temporally align the three systems (GE scanner, RPM camera, and VisionRT camera), we matched the X‐ray on/off pulses of the RPM output file (rpm pulse) and the Gate CT output file (gct pulse) (Fig. 2, center and lower panel, respectively) with the extracted midscan times of the CT images. Once the X‐ray on/off pulses were aligned, the amplitude waveforms from *gct_amp* and *rpm_amp* were compared with *ground truth* to compute the time delay.

### B. Experiment 2: Resorting and reconstruction

**Figure 3:**
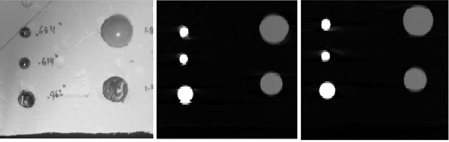
Scanned object for evaluation of 4D volume reconstruction. The middle and the right panel refers to RPM and GCT reconstruction, respectively (exhale phase).

For the second test, we coupled the mechanism shown in Fig. 1 with a box including five spheres (see Fig. 3) to generate 3 cm periodic motion along the superior–inferior direction. As in Experiment 1, RPM and GCT were used simultaneously to record the anterior–posterior motion of the plate.

The parameters used during image acquisition were the same that are used for clinical routine: image reconstruction on a full gantry rotation (0.5 sec), 0.35 sec as cine time interval and 2.5 mm as slice thickness, 8 slices per couch position (cp). The motion period was set to 3 sec. The CT images were retrospectively binned into 10 volumes (corresponding to 10 respiratory phases) using our standard clinical protocol, which is a phase‐based resorting using the GE Advantage 4D software (GE Healthcare, Waukesha, WI). Resorting was done separately for the RPM and GateCT surrogates, using waveforms produced by each system (Fig. 3 central and right panel, respectively).

The five spheres were automatically contoured on each of the 10 phases using a fixed threshold which was chosen to match the true object volume on a separate static CT scan. Spheres 3, 4 and 5 were made of the same material and the same threshold was selected. The volume of each sphere was computed using a specific tool of the GE Advanced Sim4 software, while the centers of mass (CM) were calculated by exporting and processing the DICOM RT contours.

### C. Experiment 3: Light interference

In addition to the geometric tests, we performed repeated scans using both the RPM and GateCT systems operating simultaneously, to test for light interference. Our center plans to use these devices simultaneously in order to (a) allow for a smooth transition from RPM to GateCT, and (b) perform head‐to‐head comparisons. Because both systems use controlled lighting for optical sensing, there is the possibility that the GateCT lighting will interfer with our existing RPM system. This test consisted of performing three scans, and observing the tracking results on the RPM system and identifying any tracking problems that might occur.

## III. RESULTS

### A. Experiment 1

**Figure 4:**
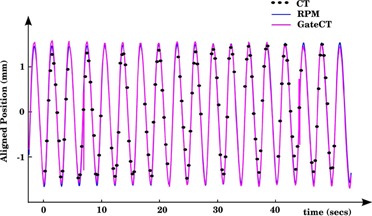
Comparison of RPM and GCT motion amplitude vs ground truth after removing the clock offset. Amplitudes were aligned by their respective mean value.

Figure 4 shows the three motion signals after time alignment. The peak of cross‐correlation was found at 0 milliseconds both for rpm‐amp/ground truth and for gct‐amp/ground truth. Because the sampling rate used for the cross‐correlation measurement was 30 Hz, the precision of this estimate is a time delay of 0±33 milliseconds. Only small differences on the order of 10 milliseconds (see Table 1) in X‐ray pulse width can be appreciated. This result quantifies the accuracy of the Blackcat GM‐10 device in synchronizing the X‐ray pulse with GCT. It should be noted that GateCT can be also electrically synchronized with the GE scanner. However, to perform a multisensor analysis we chose the option to use an external radiation detector.

**Table 1:** X‐ray pulse width for the three systems, mean (std) [sec]. Data are averaged over nine couch positions.

*Scanner Pulse [sec]*	*rpm Pulse [sec]*	*gct Pulse [sec]*
4.3999	4.4045	4.4612
(0)	(0.0005)	(0.0160)

### B. Experiment 2

Table 2 reports the results of the spheres' thresholding on the static scan. Residual errors are in <0.7% of the original volume, and these are mainly due to partial volume effects, slice‐interpolation and contours extraction.

**Table 2:** Selected threshold for each sphere and comparison between computed and real volume.

	*Sphere 1*	*Sphere 2*	*Sphere 3*	*Sphere 4*	*Sphere 5*
Threshold [HU]	−420	−570	−850	−850	−850
Ground Truth [${\text{cm}}^{3}$]	54.95	25.09	7.64	1.99	2.19
Computed Volume [${\text{cm}}^{3}$]	55.00	25.10	7.60	2.00	2.20
Computed vs. Ground Truth [${\text{cm}}^{3}$]	−0.05	−0.01	0.04	−0.01	−0.01

In Table 3, the comparison between RPM and GCT for image sorting is shown in terms of volumes of the spheres. For both RPM and GCT, discrepancies of reconstructed volume with respect to ground truth were found. This is principally due to the large range of motion and to the short breathing cycle.

**Table 3:** Real and estimated volume after image sorting. Volume values and discrepancies were averaged on 10 phases, mean (std)[${\text{cm}}^{3}$].

	*Sphere 1* [${cm}^{3}$ *]*	*Sphere 2* [${cm}^{3}$ *]*	*Sphere 3* [${cm}^{3}$ *]*	*Sphere 4* [${cm}^{3}$ *]*	*Sphere 5* [${cm}^{3}$ *]*
Ground Truth	55.0	25.1	7.6	2.0	2.2
RPM	51.8 (3.0)	24.7 (3.3)	7.7 (0.8)	1.8 (0.3)	2.0 (0.3)
GCT	51.7 (2.9)	23.6 (2.0)	8.0 (1.0)	2.0 (0.2)	2.0 (0.3)
RPM vs Ground Truth	‐3.1 (3.0)	‐0.4 (3.3)	0.0 (0.8)	‐0.2 (0.3)	0.2 (0.3)
GCT vs Ground Truth	‐3.3 (2.9)	‐1.5 (2.5)	0.4 (2.0)	‐0.1 (0.2)	0.2 (0.3)

A more detailed analysis of RPM vs. GCT per phases is presented in Table 4. In the worst case (Phase 5) GCT rebinning led to the most accurate result compared to the ground truth ($31.7\;{\text{cm}}^{3}$ vs. $24.1\;{\text{cm}}^{3}$ for rpm and gct, respectively).

**Table 4:** Comparison in terms of volume discrepancies and CM between RPM and GCT in each phase.

*Phase*	*Sphere 1*		*Sphere 2*		*Sphere 3*		*Sphere 4*		*Sphere 5*	
	*Vol* [${cm}^{3}$ *]*	*CM [mm]*	*Vol* [${cm}^{3}$ *]*	*CM [mm]*	*Vol* [${cm}^{3}$ *]*	*CM [mm]*	*Vol* [${cm}^{3}$ *]*	*CM [mm]*	*Vol* [${cm}^{3}$ *]*	*CM [mm]*
1	0.0	0.0	0.0	0.0	0.0	0.0	0.0	0.0	0.0	0.0
2	0.0	0.0	0.0	0.0	0.0	0.0	0.0	0.0	0.0	0.0
3	0.0	0.0	−0.8	0.7	0.1	0.2	−0.4	1.3	0.0	0.0
4	0.0	0.0	−2.4	2.9	0.0	0.0	0.8	2.5	0.0	0.0
5	0.0	0.0	−7.6	4.8	0.0	0.0	0.2	1.0	0.0	0.0
6	0.0	0.0	0.0	0.0	3.4	5.3	0.0	0.0	0.0	0.0
7	0.0	0.0	0.0	0.0	0.0	0.0	−0.2	0.7	0.0	0.0
8	−0.8	0.1	0.0	0.6	0.0	0.3	0.0	0.0	0.0	0.0
9	0.0	0.0	0.0	0.0	0.0	0.0	0.0	0.0	0.0	0.0
10	−0.3	0.0	0.0	0.0	0.0	0.0	0.6	2.3	0.0	0.0
mean	−0.1	−0.1	−1.1	−1.1	0.4	0.4	0.1	0.1	0.0	0.0
std	0.3	0.3	2.4	2.4	1.1	1.1	0.4	0.4	0.0	0.0

Table 4 also shows the Euclidean distance between CMs computed on RPM‐based and GCT‐based volume resorting. For this measurement, we could not compare against ground truth, so we only made a relative comparison of the two systems. In most of the cases, we found CM discrepancies when differences in volume were also detected. However, in some cases, the CM results are more variable compared to the volume. This may be due to the fact the volumes were calculated by means of the GE Advantage Sim4D software, while CMs were computed on from independent software using the DICOM RT files.

**Figure 5:**
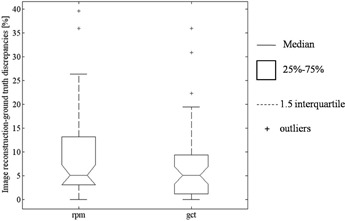
Relative error in volume reconstruction for both RPM and GCT sorting ((1‐RPM/GroundTruth)%) and (1‐GCT%/ GroundTruth)%).

Figure 5 shows the discrepancies in percentage between RPM/GCT and ground truth. Although a larger error variability was found for RPM image sorting −5%±10% vs. 5%±8% for RPM and GCT, respectively (median±quartile) – the Kruskall‐Wallis test did not proved statistical difference between the two systems.

### C. Experiment 3

We visually observed the tracking performance of the RPM system during three 4D CT scans while the GateCT system was actively acquiring data. No loss of tracking was observed in any of the three scans.

## IV. DISCUSSION

The results of our present study indicate that GateCT was performing properly, and suggest similar performance to the RPM system. However, we wish to point out some limitations of our study and suggest areas for further exploration. The most important limitation is that our evaluation was based on phantom measurements with regular motion. It is well known that many patients exhibit irregular breathing, and that irregular breathing causes artifacts in 4D CT. This problem is not unique to the two surrogates investigated here, and we feel it is unlikely that a surface‐based camera will have any particular advantage in solving this problem.

A related, but distinct problem is in recovering the phase‐shift between internal motion and the motion of an external surrogate. It is possible that surface‐based imaging could be better than measuring a single point such as RPM, or measuring tidal volume such as spirometry. For example, by carefully choosing points of interest on the patient surface, we may be able to distinguish between chest‐breathing or abdomen‐breathing. As well, we believe the additional surface data might be useful for finding improvements in phase‐based and amplitude‐based rebinning. These are important topics for future studies.

Another limitation of this study is the use a flat surface for motion tracking. Because the surface imager averages the position over a region of interest (e.g., 3 cm× 3 cm), it is possible that the rounded surfaces of some patients will cause a problem in tracking. Furthermore, because our phantom only moves the surrogate in the AP direction, we could not evaluate the system for non‐AP motion.

In Experiment 2, we report discrepancies between volumes reported by both systems, and ground truth volumes as measured using calipers. Difference in volume reconstruction can be possibly explained by the following reasons:

1. RPM and GateCT softwares use a different algorithm for assigning phase values to amplitude.
2. Since RPM and GateCT have different sampling rate (30 Hz and 16 Hz, respectively), phase reconstruction is affected by different amplitude sampling.

These errors might be reduced by increasing the temporal sampling rate of GateCT system, and perhaps also by using finer slices. In our study, we consider only the evaluation of these systems under our current scanning protocol. Optimizing the scanning protocol is an area of future work.

There are some limitations of the GateCT device that we noted. As seen in Fig. 2, the current generation GateCT device suffers from occasional, momentary loss of track. This might be due to occlusion, cast shadows or insufficient light strength. The device is ceiling‐mounted rather than couch‐mounted, and is angled low so as to be able to see into the CT bore. As a result, portions of the patient chest cannot be seen clearly due to occlusion. Furthermore, the ceiling‐mounted system must compensate for couch motion using software, which means that the respiratory waveform is not reliable during couch motion. In the worst case, patient re‐irradiation may be required to obtain comprehensive samples, thus delivering undesired dose. Finally, GateCT sampling frequency depends on the size of the tracked surface patch. If the region of interest is enlarged too much, the sampled dataset may result limited, leading to uncorrected image rebinning.

## V. CONCLUSIONS

The goal of this study was to evaluate a new optical device for 4D CT reconstruction prior to introduction into the clinic. The study assessed the consistency of GateCT and RPM in synchronizing simultaneously the respiratory signal with the image acquisition. On the basis of these results, we have confidence in the simultaneous use of these two systems for patient studies. Some discrepancy in image sorting underlines the necessity to set up additional phantom experiments for correction of phase detection. In this framework, the potential of the GateCT device can be explored by selecting different respiratory surrogate points on the surface, or using the whole surface itself as a guide for volume reconstruction.

## ACKNOWLEDGMENTS

This work was supported in part by the “Progetto Rocca” Foundation, a collaboration program between MIT and Politecnico di Milano.
